# Quantum Biology and the Potential Role of Entanglement and Tunneling in Non-Targeted Effects of Ionizing Radiation: A Review and Proposed Model

**DOI:** 10.3390/ijms242216464

**Published:** 2023-11-17

**Authors:** Bruno F. E. Matarèse, Andrej Rusin, Colin Seymour, Carmel Mothersill

**Affiliations:** 1 Department of Haematology, University of Cambridge, Cambridge CB2 1TN, UK; bfem2@cam.ac.uk; 2 Department of Physics, University of Cambridge, Cambridge CB2 1TN, UK; 3 Department of Biology, McMaster University, Hamilton, ON L8S 4L8, Canada; rusina@mcmaster.ca (A.R.); seymouc@mcmaster.ca (C.S.)

**Keywords:** quantum biology, non-targeted effects (NTE), bystander effect (RIBE), stressors, environmental radiation exposure, cellular communication, quantum information, quantum physics, hormesis, adaptive response

## Abstract

It is well established that cells, tissues, and organisms exposed to low doses of ionizing radiation can induce effects in non-irradiated neighbors (non-targeted effects or NTE), but the mechanisms remain unclear. This is especially true of the initial steps leading to the release of signaling molecules contained in exosomes. Voltage-gated ion channels, photon emissions, and calcium fluxes are all involved but the precise sequence of events is not yet known. We identified what may be a quantum entanglement type of effect and this prompted us to consider whether aspects of quantum biology such as tunneling and entanglement may underlie the initial events leading to NTE. We review the field where it may be relevant to ionizing radiation processes. These include NTE, low-dose hyper-radiosensitivity, hormesis, and the adaptive response. Finally, we present a possible quantum biological-based model for NTE.

## 1. Introduction

Non-targeted effects (NTE) are an intriguing phenomenon where the biological responses observed in cells or tissues are not directly exposed to a stressor (e.g., ionizing radiation or chemical agents). These effects can occur as a result of signaling and communication between exposed and non-exposed cells, leading to alterations in cellular behavior and function beyond the irradiated or exposed region [1]. NTE have implications for various fields, including radiation biology, toxicology, and environmental health, which demonstrates that it is essential to consider them in understanding the broader impact of stressors on biological systems [2,3]. Traditionally, the harmful effects of ionizing radiation have been attributed solely to the direct damage caused by energy deposition in the target cells [4,5]. However, research in recent years has revealed that radiation-induced effects extend beyond the directly irradiated cells, impacting neighboring and distant cells that are not exposed to radiation [6,7]. These NTE can result from signaling mechanisms, such as bystander effects and genomic instability, which trigger cellular responses in non-irradiated tissues [4]. They can also result from hormesis mechanisms, discussed later, which, in common with NTE, involve reactive oxygen species, bioelectric effects in mitochondria, and a number of epigenetic mechanisms, including genomic imprinting and methylation [8].

NTE have considerable significance in radiation exposure, including medical radiation therapy, environmental radiation exposure, and occupational exposures [9].

Understanding these effects takes center stage in the realm of optimizing radiation treatment strategies. The goal here is to strike a balance—maximizing the impact on cancer cells while minimizing any collateral damage to healthy tissues [10]. In environmental and occupational settings, the presence of NTE indicates that exposure to ionizing radiation or other potentially harmful agents can lead to effects not only at the site of exposure but also in distant and seemingly unexposed tissues [11]. Increasing evidence is also emerging for the interorganism and even interspecies communication of signals, leading to effects in unexposed individuals [12]. This potentially provides a bridge allowing population-level effects to be recorded. Current radiation protection regulations primarily focus on the direct effects of radiation on the target tissues [13,14]; however, the effects of radiation exposure are complex. Not all effects are harmful and there are many documented reports of low-dose hormesis and induced adaptive responses. Low-dose hypersensitivity has also been reported both in vivo and in vitro, meaning that it is critical to understand how and why a particular response dominates. Since NTE mechanisms and responses are so important in low-dose radiobiology, incorporating NTE into radiation protection models can lead to more comprehensive and accurate assessments of radiation risks to ensure better protection for individuals and populations [15,16].

**Figure 1:**
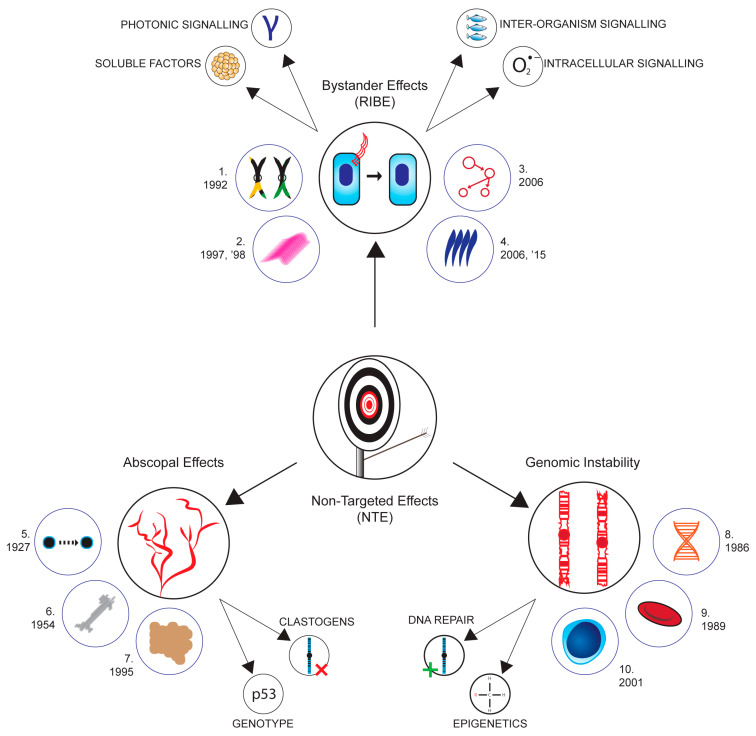
State of the art in non-targeted effects (NTE) research. Illuminating the landscape of non-targeted effects (NTE) research. Exploring bystander effects (e.g., cellular signaling, photonic signaling, interorganism signaling, and soluble factors), genomic instability (including the positive action of DNA repair mechanisms (green plus) and epigenetic changes), and abscopal effects with the negative impact of clastogens (red cross). 1. [39]; 2. [40,41]; 3. [42]; 4. [43,44,45]; 5. [46]; 6. [47]; 7. [48]; 8. [49]; 9. [50]. 10. [51].

Despite the significant progress made in unraveling the complexities of the non-targeted effects (NTE) induced by ionizing radiation, there are still several limitations in our current knowledge of the underlying mechanisms [16,17,18]. One major challenge is the multifaceted nature of NTE, which involve signaling pathways, cellular responses, and intercellular communication processes that extend beyond the directly irradiated cells [19]. The exact molecular events that trigger and propagate these non-targeted responses are becoming better understood and involve photon emission, the exosome-mediated transfer of information, the elevation of reactive oxygen species, and ion channel perturbations in the irradiated entities [20], while, in the recipients, TGF-beta, p53, and many other stress response and DNA damage response pathways are activated [21]. Mitochondria play a major role in the NTE processes (see the comprehensive review by Averbeck, 2023 [22]). Figure 1 shows the current state of the art in the field.

Living systems fundamentally rely on electrical processes (e.g., biochemical reactions or the flow of ions across cellular membranes) [23], and the energy transfer within these systems occurs through various mechanisms involving electromagnetic radiation (e.g., photons) and electric field fluctuations (e.g., electron interactions with photons) [24,25]. These processes encompass excitation and ionization, as well as rotational and vibrational transitions [26,27], and have the potential to induce damage and disrupt the normal functioning of molecules and biological processes, which may lead to various consequences, including the formation of free radicals, the activation of enzymes, protein malfunction or structural alterations, and changes in gene expression [28,29]. In the 1970s, David DeVault and Britton Chance made significant contributions to our understanding of these biological processes [24]. DeVault focused on molecular dynamics, electronic energy transfer, and reaction mechanisms in biological systems, especially electron transfer reactions in photosynthesis [24]. Britton Chance, on the other hand, concentrated on enzyme kinetics and pioneered the use of NADH as a marker for mitochondrial function. He developed non-invasive techniques to study mitochondrial redox states and advanced spectroscopic methods, expanding the applications of spectroscopy in enzyme kinetics and bioenergetics. Furthermore, for over a century, morphogenetic and bioelectric fields have been used to understand life processes [30,31]. Morphogenetic fields organize biological form development [32], while bioelectric fields result from ion movement across cell membranes and influence various cellular processes. These fields may be influenced by quantum effects [32], where entanglement facilitates long-distance communication between morphogenetic fields. This quantum phenomenon, coupled with tunneling activates signaling pathways within bioelectric fields. Fritz-Albert Popp significantly contributed to the study of biophotons [33,34], exemplified by his development of a photon counter, an instrument capable of quantifying biophoton emissions from living organisms. This helps us understand how biofields and biophotons are connected, revealing their quantum foundations. Popp’s investigations revealed that a variety of stimuli, including stress and radiation exposure, could stimulate an increase in biophoton emissions [35,36,37]. Biophotons, ultraweak photons emitted by living organisms, have been observed across a range of wavelengths, from ultraviolet (UV) to infrared (IR) [38], documented across various life forms, from bacteria to humans. While the exact mechanisms behind biophoton production are under ongoing research, they are believed to be associated with vital cellular processes such as metabolism, cell signaling, and DNA repair.

Conventional radiation biology approaches have predominantly focused on the macroscopic effects of ionizing radiation, overlooking the quantum-scale interactions that may play a crucial role in NTE [16,52,53]. Quantum biology (QB) offers a unique perspective to explore and understand the intricate and subtle processes underlying NTE [54,55]. In fact, the quantum effects hold the promise of understanding the mechanisms that govern NTE at the molecular and subcellular levels, offering tools that enable us to investigate how quantum processes shape interactions between molecules, signaling pathways, and cellular functions, as well as shedding light on the intricate dynamics underlying NTE responses and opening new avenues in radiation biology. As a result, more effective therapeutic approaches, improved radiation protection standards, and a better understanding of the effects of ionizing radiation on human health can be developed [52,56,57].

## 2. Non-Targeted Effects (NTE) Gaps in Our Knowledge

**Figure 2:**
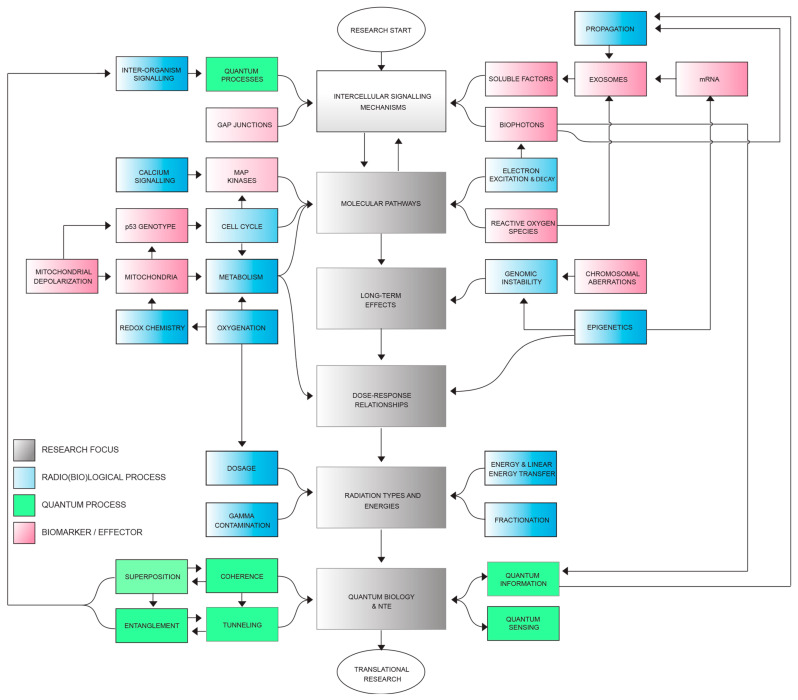
Knowledge gaps in non-targeted effects (NTE) of radiation. This flowchart illustrates critical knowledge gaps in NTE research, emphasizing the influence of quantum biology. It highlights areas demanding deeper exploration, ultimately advancing NTE understanding within radiation biology, therapy, and related domains, while suggesting potential links to quantum phenomena like coherence, entanglement, and tunneling as pivotal initiators of NTE.

Despite the considerable progress in our understanding of NTE, significant gaps in our knowledge remain (see Figure 2). For example, the long-term consequences of NTE on the cellular and organismal levels are not well established and the persistence and impact of NTE-induced alterations over time could lead to genomic instability and an increased risk of developing deleterious mutations or abnormalities [58,59,60]. On the other hand, they could lead to beneficial effects such as hormesis, which, at the population level, could result in adaptive evolutionary progress. Long-term effects understanding is therefore vital in the context of radiation therapy, environmental radiation exposure, and potential long-term health risks [61,62]. Additionally, the dose–response relationships for various types of NTE are not fully understood as they frequently demonstrate nonlinearity and appear to dominate the low-dose response [63,64]. Differentiating the effects of various radiation doses on NTE could be critical in optimizing radiation therapy protocols and assessing the potential risks or benefits associated with environmental radiation exposure [65,66]. Furthermore, it is crucial to investigate how different types of ionizing radiation (e.g., X-rays, gamma rays, neutrons, protons, and alpha particles) and their energies influence NTE responses [67]. While there is a lot of work published in this field, there are significant anomalies, such as the fact that neutrons (where gamma contamination of the beam is totally shielded) do not seem to produce a bystander effect or genomic instability [68,69,70]. Another issue is that alpha particles produce genomic instability but the evidence for the induction of bystander signaling is contradictory and controversial [71].

Another significant gap is in the complex interactions between cells within tissues and organs, which can influence NTE responses [72,73,74]. In complex multicellular organisms like humans, cells are intricately organized into specialized tissues that collaborate to fulfill distinct roles. Organs, composed of multiple tissues, are organized to perform specific tasks. Connective tissues, which are made up of an extracellular matrix, living cells, and a nonliving component known as ground substance, are essential for connecting cells and organs, as well as providing their protection, support, and integration. This complex interplay of different cell types, each with its own role within tissues and organs, unravels how these intricate interactions impact NTE, the understanding of which is challenging due to the complexity of multicellular systems [75,76]. Understanding the role of the tissue microenvironment and cellular communication in NTE [77,78] requires the exploration of quantum effects during the initial events leading to NTE [79,80,81].

Other areas where the consideration of quantum effects could be important include how photons are produced, and whether they exist solely in the UVA range or span across the spectrum. Moreover, why do UVA photons play a pivotal role in modulating NTE? [79,82,83]. Ion-gated channels are known to be important, but are calcium channels the sole players, or do sodium and potassium channels also contribute? The interaction between photons and mitochondria could also involve quantum biology, as discussed later, particularly in terms of how the photons block complex 1 activity and which part of complex 1 holds the key to this process. Exosome formation and cargo control are also interesting, with questions emerging about the content selection process and how the irradiated cell determines what goes into the exosome [84,85,86]. Equally intriguing is the regulation of exosome shedding and the identity of receptors on unirradiated cells that receive the exosome cargo, along with how the cargo is processed to activate recipient cell responses [87,88,89]. Are these processes uniform across diverse organisms, such as fish, frogs, rodents, and crickets? Lastly, the question of why some cell types fail to produce or respond to signals is unresolved.

Addressing these questions, which typically involve early electrical or mitochondrial events with suspected quantum processes, is critical to improve our understanding of NTE and their far-reaching implications for radiation biology and therapy. Despite our understanding of NTE in a variety of organisms, such as fish, frogs, rodents, and crickets, the biologically distinct responses and interactions remain a mystery, and understanding these inconsistencies is critical in predicting the potential impact of radiation exposure on various ecosystems and their inhabitants, as well as guiding environmental and radiation safety measures.

## 3. Quantum Biology and NTE

### 3.1. Quantum Physics Meets Biology

Quantum physics, often referred to as quantum mechanics, explores the behavior of matter and energy at the most minuscule scales, encompassing the behavior of atoms and subatomic particles (e.g., electrons, protons, and photons) [90,91,92]. It stands apart from classical physics by allowing particles to exist in multiple states simultaneously—a concept known as wave–particle duality. This fundamental shift in perspective emerged in the early 20th century, addressing previously unexplained phenomena like the photoelectric effect and the blackbody spectrum [93,94,95,96]. Wave–particle duality, a central concept in quantum physics, reveals that particles can exhibit both wave-like and particle-like properties [97]. This duality is exemplified in the double-slit experiment [98,99], where electrons fired at a double slit create interference patterns akin to waves. While proposed a century ago, wave–particle duality remains crucial in understanding the behavior of matter at atomic and subatomic scales.

Quantum biology, which emerges at the nexus of quantum physics and biology, aims to explore the potential influence of quantum phenomena within biological processes, from the molecular to the organismal [79,80,81]. The terms “quantum effects”, “quantum behavior”, and “quantum phenomena” are related terms in quantum mechanics, describing the behavior of particles and systems at the subatomic level, such as electrons, photons, atoms, and molecules. Quantum effects manifest in various biological phenomena (see Section 3.2), affecting the timing and efficiency of essential biochemical reactions [100,101]. The extent of these quantum effects within the intricate realm of biology remains a challenging question [32,57]. Navigating quantum biological scales presents a formidable challenge in understanding the precise scope of quantum effects within biology. These effects are established at molecular and cellular scales, but evidence suggests their involvement even in the realm of organisms (e.g., intriguing research indicates that birds might leverage quantum effects in their remarkable navigation abilities) [102]. Quantum biology challenges long-held beliefs that limit quantum effects to microscopic scales within the warm and wet conditions of life and pushes the boundaries of our understanding of biology by examining how quantum effects, once thought to be relevant only at the microscopic scale, can play a role in complex, macroscopic biological systems [57]. Traditionally, physicists assumed that high temperatures, a low vacuum, or strong interactions in living cells limited the existence of quantum effects. A paradigm shift suggests that quantum effects can indeed operate within the complex fabric of biological systems [103,104]. However, a fundamental question persists—to what extent do these quantum effects stretch in time and space within the intricate tapestry of biology?

Classical and quantum variability, precisely the stochastic behaviors of their fluctuations, are crucial in understanding the interplay of biological systems exposed to radiation. Classical fluctuations are driven by various sources, including thermal motion, molecular interactions, and external factors such as radiation, where the stochastic behavior of these classical fluctuations arises from the random and chaotic movement of particles at the macroscopic level [23,105]. Due to their large-scale nature, classical fluctuations can often be described statistically, with behaviors following Gaussian or normal distributions. These fluctuations exhibit a degree of predictability when analyzed collectively but remain inherently stochastic at the individual particle level. In the context of radiation exposure, classical fluctuations can lead to unpredictable variations in tissue temperature, the generation of reactive oxygen species (ROS), which include molecules like superoxide (O_2_^•−^) and hydrogen peroxide (H_2_O_2_), and subsequent cellular responses, all of which are characterized by random and fluctuating patterns. Quantum fluctuations, on the other hand, are inherently stochastic at a fundamental level due to the Heisenberg Uncertainty Principle [106,107]. They are minute random fluctuations in the values of the fields that represent elementary particles, such as electric and magnetic fields, which represent the electromagnetic force carried by photons. This principle states that it is impossible to precisely determine both the position and momentum of a particle simultaneously. The energy of an electron in an atom is not constant, but rather fluctuates up and down slightly. As a result, quantum fluctuations arise due to the inherent uncertainty in particle behavior at the subatomic level. These fluctuations manifest as unpredictable variations in the positions, momenta, and energies of quantum entities. The stochastic behavior of quantum fluctuations is a fundamental aspect of quantum mechanics and is particularly pronounced in the microscopic world of biological molecules and cellular components. It introduces inherent randomness and indeterminacy into processes such as DNA repair, enzyme catalysis, and cellular signaling [103,108].

Biophotons are photons emitted by living organisms and can be classified into classical and quantum based on the processes through which they are emitted, absorbed, or transmitted. Classical biophotons are emitted by random, spontaneous transitions of biological molecules from one energy state to another and are not entangled or do not exhibit quantum coherence. Quantum biophotons, on the other hand, are emitted through quantum processes, such as resonant energy transfer or quantum tunneling, and can be entangled or exhibit quantum coherence [104]. The distinction between classical and quantum biophotons is not always clear-cut and, in many cases, biophotons may exhibit both classical and quantum properties (e.g., even if emitted through a quantum process, they may still exhibit random fluctuations in timing and intensity),whose significance for quantum fluctuations becomes particularly pronounced in the bystander effect (e.g., entangled biophotons emitted by a cell could convey information about radiation damage to other cells in the body, even if not directly targeted by radiation) [109]. Moreover, quantum fluctuations in biophotons may influence cellular responses to radiation damage and the potential for their quantum coherence is imbued with quantum-encoded information [110], which influence gene expression and various cellular processes, ultimately contributing to the bystander effect [111]. When emitted by irradiated cells, they may act as signaling agents, affecting gene expression and other cellular processes. Understanding the role of quantum effects is crucial in mediating the non-targeted effects of ionizing radiation on processes influenced by infrared radiation (IR) [112]—specifically, how quantum entanglement can facilitate IR-mediated signaling across long distances and how quantum tunneling can enable IR-mediated energy transfer even through barriers [113,114].

**Figure 3:**
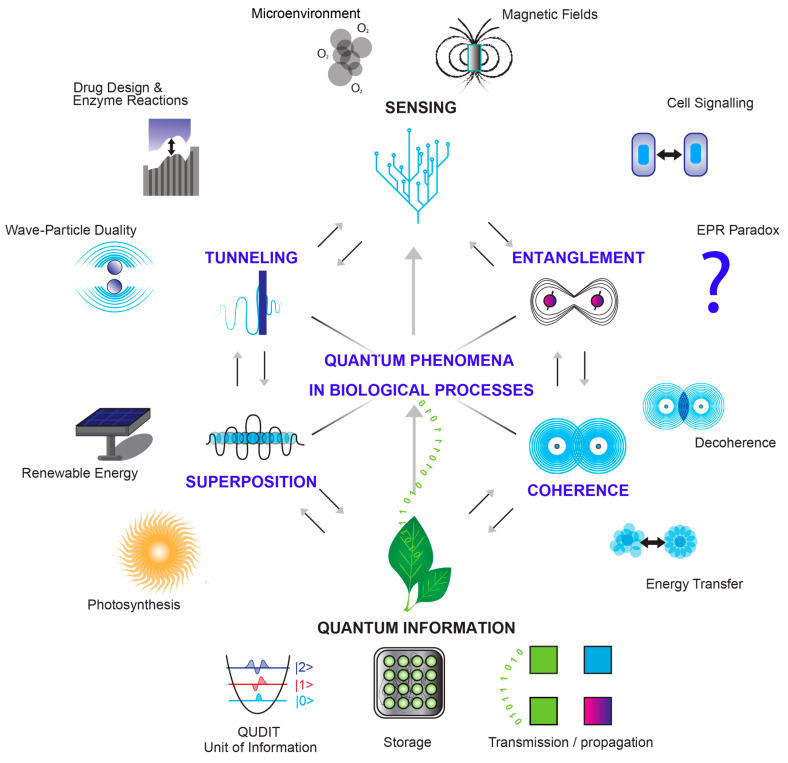
Quantum phenomena in biology. Quantum superposition—revealing simultaneous particle states: promising enhanced light harvesting in photosynthesis and pioneering renewable energy applications. Quantum entanglement—illustrating correlated states: significance in cellular signaling and the EPR paradox’s interconnected particles which raises deep questions (symbolized as an interrogation mark) about the nature of reality, the role of observation, and the fundamental principles of quantum mechanics. Quantum tunneling—visualizing barrier overcoming: enzymatic reaction applications and wave–particle duality. Quantum coherence—depicting maintained phase relationships: explore cellular energy transfer and function impact, and coherence vs. decoherence in quantum systems. Quantum sensing—demonstrating signal detection: encompasses cellular environment sensing and magnetic field detection in biology. Quantum information—unleashing biological data power: ‘Qdits’ in d-dimensional complex vector spaces for the representation of a complex unit of biological data that could be observed during manipulation, storage, and transmission/propagation.

Quantum entities, such as electrons, protons, and photons, are fundamental to biological processes. They exhibit both particle-like and wave-like behaviors, famously experimented with the double-slit experiment. Electrons, despite their negative charge, are central to energy generation within cellular respiration and metabolic processes [115,116,117,118]. Their unique feature is the wave–particle duality, allowing them to simultaneously exist as both discrete particles, participating in chemical reactions, and as quantum entities exhibiting wave-like properties. This duality enables phenomena like quantum tunneling, which plays a pivotal role in energy and information transfer within living organisms. Protons, positively charged particles found in atomic nuclei, have precise roles in regulating the pH balance and enzymatic activity [119,120]. Just like electrons, they exhibit similar wave–particle duality in the quantum context, influencing energy transfer reactions and chemical interactions in biological systems [121,122]. Finally, photons are carriers of light and electromagnetic radiation, essential in various biological processes, including photosynthesis, vision, and medical imaging [123,124]. Furthermore, Marcus Arndt’s work in matter wave interferometry demonstrates that not only electrons and photons but also relatively larger molecules, such as those found in biological systems (e.g., protoporphyrins), can exhibit wave–particle duality effects and therefore display quantum behaviors, such as quantum tunneling, coherence, and entanglement, and these effects impact the biological domain (see Figure 3) [57,125].

- Quantum superposition

The concept of quantum superposition refers to the remarkable ability of tiny particles to exist in multiple states simultaneously [126], akin to simultaneously juggling multiple tasks [127,128,129]. This concept is relevant in various biological and physical processes, including the intriguing phenomenon of photosynthesis (e.g., how plants and bacteria utilize sunlight for energy production) [80,130]. In non-classical physics, particles have the potential to occupy different states simultaneously, much like plants capturing sunlight from multiple angles to drive energy production [80,130], which offers practical implications in enhancing renewable energy technologies (e.g., efficient solar panels by enabling them to capture more sunlight and convert it into energy more effectively) [131,132]. To grasp this concept more comprehensively, let us consider the notion of “quantum states”, where there are various ways in which particles can arrange themselves [133,134,135,136]. These states are described using “quantum numbers” [137], which act as labels explaining particle behavior. To illustrate, imagine a spinning top that can rotate in two directions—particles like electrons possess a spin, either “up” or “down”, [138,139,140,141], characterized using quantum numbers like “n” and “m”. When these quantum states interact, they give rise to new combinations, much like mixing colors to create new shades. This phenomenon can be described as particles behaving like waves, which produce different patterns as their states merge [142,143,144]. An analogy could be drawn from water waves overlapping to form intricate patterns. Particles have in fact intriguing wave characteristics when their quantum states come into contact, resulting in fascinating effects. This can be considered as putting together puzzle pieces to reveal the entire picture—the collective quantum states form the entire system, just like fitting puzzle pieces to create a complete image, where each observable quantum state is made up of smaller component states [133,134,145]. Finally, the Schrödinger equation, a mathematical tool, plays a pivotal role in elucidating how particles manifest wave-like properties [94,95,96], which enables us to simulate how particles respond when they interact with their environment [126].

- Quantum Entanglement

Quantum entanglement is a mysterious phenomenon where two or more quantum systems are interconnected in such a way that they share the same properties, even when separated by vast distances. In this context, when two or more particles become entangled, their states become correlated, regardless of distance, which could potentially influence cellular signaling and communication processes. Einstein’s term, “spooky action at a distance”, captures its puzzling nature, as it seems to defy the laws of classical physics. Nevertheless, this phenomenon has been repeatedly confirmed through experiments. It refers to the interactions of particles whose states cannot be independently described accurately, even when separated by considerable distances [146,147,148]. This intriguing phenomenon has sparked debates due to its unique properties (e.g., measuring one entangled particle directly affects the others, leading to seemingly paradoxical effects), and the Einstein–Podolsky–Rosen (EPR) Paradox serves as an example of the complexity and implications of entanglement [149,150]. Interestingly, pairs of entangled electrons in “Posner molecules”, clusters of phosphate and calcium ions, have been shown to potentially transfer energy between cells. Moreover, in the development of the nervous system, the coordinated activity of neurons is crucial. Some models that use quantum-like concepts to describe neuron activity suggest that entanglement-like phenomena could play a role in the complex coordination required for nervous system development [151]. In terms of synaptic plasticity, which is the ability of synapses to strengthen or weaken over time, some studies have suggested a potential role for entanglement-like phenomena, which could potentially influence how our brains learn and adapt to new experiences [151]. Regarding brain damage repair, while there is no direct evidence linking entanglement to brain repair processes, the brain does have remarkable abilities to repair and reorganize itself, which involves processes such as neuroplasticity, where the brain forms new neural connections, and synaptic plasticity, where the strength of synapses changes. While interpretations regarding wave function collapse upon measurement and the speed of entangled particle influence may vary, physicists concur that entanglement creates correlations between measurements, carrying potential for technological applications [152,153,154,155], such as the creation of ghost images using parametric down-conversion (converting a blue photon into two red ones), which showcases the tangible significance of entanglement, highlighting its profound implications for technology and science.

- Quantum tunneling

Quantum tunneling refers to the extraordinary ability of particles to transcend barriers that should be insurmountable according to the principles of classical physics. These effects have reshaped our understanding of chemistry on a microscopic scale and hold the potential to catalyze advances in medicine and enrich our comprehension of enzymatic functions [156]. To comprehend quantum tunneling, consider quantum particles as probability waves. They can exist at various points within their wavefunctions, just like a die has the possibility of landing on any side. Even though rolling a six is statistically less likely, it remains possible. Quantum tunneling is akin to this unpredictability. Despite a quantum particle’s apparent lack of energy to cross an energy barrier, there is still a possibility that it can tunnel through because its wavefunction extends to the other side of the barrier. To further comprehend this, imagine encountering an imposing hill obstructing a path and lacking the energy to ascend it. Within the realm of quantum physics, these minuscule particles exhibit the astonishing capability to breach such barriers, akin to traversing an imperceptible barrier. In a parallel narrative, consider playing a game where a ball can occasionally traverse a wall. Similarly, “wave–particle duality” characterizes the behavior of minuscule entities [157,158,159]. Electrons, for instance, exhibit both wave-like and particle-like traits. This duality extends to light, behaving as waves or photons. This duality’s consequence is the “Heisenberg Uncertainty Principle”, introducing a range between certainty and impossibility in particle movement (such as electrons, protons, and photons) [160,161]. Electrons might transcend barriers, albeit infrequently. Understanding this quantum puzzle and its role in cellular mechanics could yield novel medical strategies and insights into enzymatic functions [162,163,164]. Enzymes, akin to microscopic machinery within our bodies, orchestrate chemical reactions. Quantum tunneling catalyzes these reactions by providing an efficient shortcut where these particles hold a hidden path that expedites reactions. Quantum tunneling has the potential to facilitate the mobility of drugs and enhance their efficacy [165].

- Quantum coherence

Quantum coherence refers to the ability of quantum systems to maintain phase relationships [148] and could play a role in various biological processes, including energy transfer within cells, potentially impacting cellular functions. These effects could facilitate energy transfer and information processing in biological systems, enhancing their adaptability and survival in changing environments [166,167,168]. However, other studies argue that coherence is fragile in warm and noisy biological conditions, and classical mechanisms can explain observed phenomena without invoking quantum effects [148,169]. Entanglement is in fact related to the ideas of quantum coherence and decoherence [147]. In classical physics, two waves are coherent if their properties produce stationary interference, but the same applies to wave functions. This can be illustrated in the already mentioned double-slit experiment, where electrons pass through both slits and form an interference pattern on a target. The coherence of the waves allows the electrons to interfere with each other [150], and, when quantum systems interact with the environment, this coherence becomes shared and lost over time, known as quantum decoherence, resulting in the loss of quantum behavior [148,170]. Identifying quantum coherence and dynamics efficiently, given limited system access, is essential for reliable quantum applications [171,172,173]. Moreover, the question of whether quantum coherence can exist in biological organisms in vivo, such as in photosynthetic complexes or avian chemical compasses, surrounded by hot and wet environments, has sparked interest in understanding the relationship between quantum coherence and biological function [174,175]. In such cases, full-system access is often limited, and the detection of the signatures of quantum coherence is often indirect.

- Quantum sensing

Quantum sensors have the potential to revolutionize our understanding of radiation-induced effects on biological systems [176,177] by providing more accurate and sensitive measurements of ionizing radiation, leading to safer and more effective treatments in medical radiology and radiation therapy [178]. Quantum sensors can also detect early cellular changes caused by radiation exposure, enabling the better monitoring of radiation-related health risks. Quantum-enhanced medical imaging techniques, such as quantum MRI and MEG, can significantly improve the quality and precision of medical imaging, providing insights into radiation-induced effects on tissues and organs [179]. Additionally, quantum biosensors can be used to detect biomarkers related to radiation exposure and its effects, such as DNA damage and repair [180]. This information can be invaluable in radiation research and occupational health monitoring. Finally, quantum-enhanced microscopy techniques [181,182] can enable the observation of biological samples exposed to radiation at the molecular and cellular levels with unprecedented detail, providing insights into the mechanisms of radiation-induced damage and repair processes in biological systems.

- Quantum information in quantum biology and radiation effects

Classical information is encoded in binary bits, typically represented as 0 or 1, while quantum information thrives on the unique property of quantum states that can exist in superpositions. Quantum information is found where the intricate behaviors of waves and particles governed by quantum mechanics are used to encode, process, and propagate data [183,184,185] or when itis associated with secure communication, precise sensing, and advanced computing, and now for potential applications in quantum biology, environmental interactions, and non-targeted effects (NTE) [80,186]. Its use in biology reveals the behavior of biomolecules and cellular systems and functions by harnessing their particle quantum properties (e.g., superposition, entanglement, coherence, tunneling, and efficient quantum state propagation). This distinct property enables novel forms of communication and processing, offering promising avenues for the detection and measurement of the non-targeted effects of ionizing radiation. In NTE, quantum phenomena are believed to play a role in the complex interplay between external stressors and biological systems, leading to unexpected responses [54]. The spread of NTE effects through biophotons and other particles is particularly significant in biological communication, influencing how living organisms interact and exchange information [187,188,189,190]. Furthermore, applying quantum information principles to environmental research has the potential to significantly improve our ability to monitor and analyze ecosystems with the use of quantum-enhanced sensing techniques, which enable us to detect even the smallest changes in environmental factors with unrivalled precision and allow us to detect ecological disruptions and potential threats early on [191,192]. The intricate interplay between ecosystems and external influences, including pollutants, climate variations, and habitat modifications, can be investigated, protected, and promoted with unprecedented precision and understanding using quantum information tools.

### 3.2. Areas Where Quantum Effects May Occur

**Figure 4:**
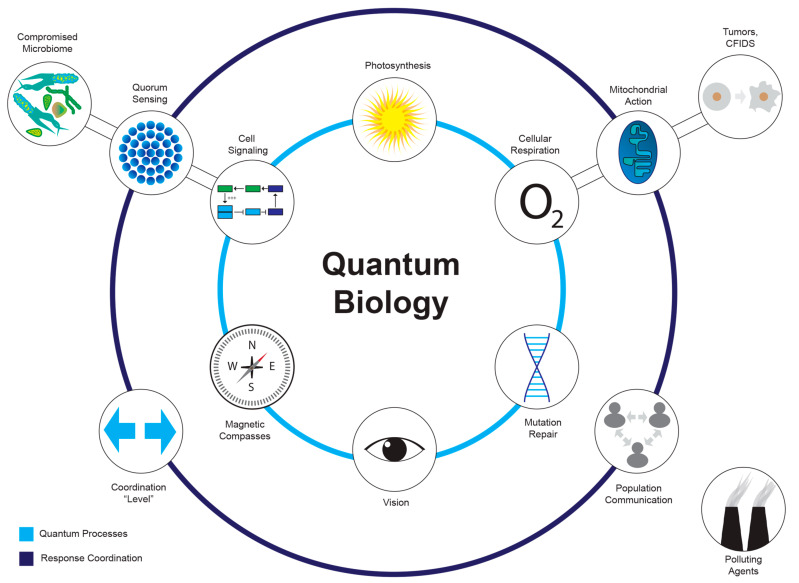
Quantum effects in biological processes. Exploring quantum influences on DNA replication and repair; cellular energy production (metabolism); cell signaling and communication; photosynthesis; enzyme catalysis; cellular communication; protein folding; and conformation.

Quantum effects have the potential to manifest in situations involving physical energy transduction, transition, or operative gradients. In essence, these effects could play a role in processes driven by electromagnetic gradients or where energy undergoes transformation, transduction, or capture (see Figure 4) [32]. Quantum effects may also influence phenomena at the intersection of morphogenetic and bioelectric fields—for instance, within the rhythmic changes at the level of gene expression, as well as protein quantities and subcellular distribution, which confer temporal features to the molecular platforms hosting electrochemical processes and non-trivial quantum phenomena [57].

1. Quantum Effects on Morphogenetic and Bioelectric Fields

In the late 18th century, Voltaire and Galvani’s early insights into the electrical nature of life formed the basis of modern research in quantum biology [193]. Their “electric fluid” concept can be seen as a precursor to the notion of quantum bioelectric fields, thought to comprise photons and other quantum particles, influencing various biological processes, such as morphogenesis, signal transduction, and nerve conduction [30]. Galvani’s experiments, demonstrating biological systems’ capacity to generate and respond to electrical signals [193], hinted at the involvement of quantum phenomena, such as entanglement, coherence, superposition, and non-locality, in bioelectric field organization and function.

Galvani’s groundbreaking experiments revealed the generation and transmission of bioelectric fields over substantial distances [109], exhibiting remarkable coherency [109], existing as both positive and negative entities simultaneously, and their susceptiblity to external stimuli like light and sound [31]. These findings imply that the particles involved in the organization and functioning of bioelectric fields may exist in an entangled state, maintain coherence, or even exist in superpositions of states, ultimately interacting non-locally with one another [57]. They also hint at the involvement of quantum phenomena in bioelectric field organization and function due to the overall complexity of the coherence of bioelectric fields, which are able to coordinate the activity of billions of cells in the body. They do this in a way that is highly efficient and precise, and this suggests that the particles involved in bioelectric field organization and function are interacting with each other in a quantum way [57].

Morphogenetic and bioelectric fields are pivotal in understanding the potential influence of quantum effects on various biological processes [194]. Morphogenetic fields are believed to guide the development of biological forms by coordinating cellular movement and tissue development. They are thought to organize the bodies of plants and animals through vibratory patterns and underlie their abilities to regenerate and heal after damage. Quantum entanglement and coherence could play a role in shaping morphogenetic fields [109], which guide biological form development through cellular coordination by influencing their organizational properties, influencing the behavior of biological molecules, or influencing the communication between cells (e.g., entangled photons could be used to control the movement of proteins and other molecules within cells) [195], and could lead to changes in cellular organization and tissue development. Furthermore, entangled particles could be used to transmit information between cells at a much faster speed than traditional chemical signaling [196], which could allow cells to coordinate their behavior more effectively and develop complex biological structures (e.g., entangled microtubules, transcription factors, immune cells, and mental information could coordinate cellular movement, regulate growth and differentiation, promote wound healing, and mediate consciousness effects on biology) [197]. Bioelectric fields are involved in various cellular processes [198,199], such as signal transduction, muscle contraction, and nerve conduction and the quantum effects such as superposition and non-locality could enhance its efficiency. Superposition might allow multiple bioelectric pathways, fostering faster nerve conduction [198]. In this scenario, a neuron could exist in a superposition of states—simultaneously firing and not firing. Such a state would empower the neuron to transmit signals to multiple targets simultaneously, allowing a significant acceleration in information processing within the brain. The bioelectric fields in different parts of the body could be correlated with each other [109], and could allow for the more efficient coordination of cellular activities. In the heart and lungs, these bioelectric fields could be correlated with each other to ensure that the two organs synchronize together efficiently to circulate blood and oxygen. The interaction of biophotons with molecules like DNA might mediate an effect called mitogenic [200], and quantum entanglement may facilitate long-distance cell communication through entangled biophotons, enabling coordinated cellular development [201].

2. Quantum Effects on Mitogenic Radiation

In the 1920s, Alexander Gurwitsch conducted groundbreaking research on photonic communication in biology [202], discovering what he termed “mitogenic radiation” [203] determined to be in the visible spectrum. Gurwitsch’s pioneering experiments involved exposing onion root tips to ultraviolet (UV) light, resulting in the emission of radiation that stimulated the growth of other onion root tips [204]. It is now widely accepted that cells can communicate using photons. Mitogenic radiation involves the emission of biophotons, which can stimulate cell growth and division [205]. These biophotons may interact with molecules like DNA, potentially influencing gene expression related to cell growth and division. Recent research on mitogenic radiation has revealed its diverse applications in medicine, spanning from cancer treatment, regenerative healing, and tissue repair to addressing neurodegenerative diseases, cardiovascular health, and skin conditions. It may stimulate normal cell growth within the tumor microenvironment [206], potentially impeding the spread of cancer cells and enhancing their sensitivity to chemotherapy and radiation therapy. Additionally, quantum biology has intriguing connections to it. Biophotons are a central element in this link, as they are involved in various cellular processes, and it is possible that the photons emitted during this radiation interact with DNA, influencing gene expression or signaling pathways (e.g., to increase the expression of genes involved in activating signaling pathways in cell growth and division). Moreover, quantum entanglement might be at play, allowing cells to communicate over long distances through the entangled emission of biophotons [201]. If one cell emits a biophoton, the other cell will also emit a biophoton, even if they are separated by a large distance. The two biophotons will be entangled, meaning that they will share the same fate. If one biophoton interacts with a molecule in the cell, the other biophoton will also interact with the same molecule in the other cell, which could allow the two cells to communicate with each other over long distances.

- Photosynthesis, vision, and magnetic compasses

Light harvesting is a process whereby pigments such as chlorophyll or rhodopsin trap sunlight and convert the radiant energy into electrical chemical energy. However, the exact quantum nature of light harvesting remains a topic of debate [103]. Cryptochromes, a class of photoreceptor proteins found in both plants and animals, contribute to these processes by generating triplet states through the absorption of photons [207,208]. These triplet states are critical in understanding the quantum aspects of photosynthesis and magnetoreception, which allows organisms to detect the Earth’s magnetic field. Experimental evidence supports the role of cryptochromes in magnetic field detection, especially in birds, which rely on these proteins for navigation. When cryptochromes are disrupted, it can lead to navigation difficulties when using the Earth’s magnetic field. Flavin adenine dinucleotide (FAD), a pivotal cofactor, plays essential roles in various biological processes [209]. It exists in two redox states, oxidized (FAD) and reduced (FADH2), and takes part in electron transfer reactions within enzymes, contributing to ATP generation through the electron transport chain. The quantum properties of FAD are closely tied to its biological functions; for instance, its ability to exist in two redox states is crucial for electron transfer reactions. Moreove, the flavin ring in FAD is associated with quantum tunneling, a process that contributes to the generation of triplet states in cryptochromes. The quantum effects responsible for energy transfer between chromophores in photosynthesis, are instrumental in energy dissipation within these biological systems. This exciton transfer play a pivotal role in safeguarding cells from damage, particularly by dissipating excess energy in the form of heat [57]. Quantum tunneling and entanglement are among the quantum phenomena that facilitate this energy dissipation in biological systems [210,211]. Recent evidence suggests that stable states lasting several femtoseconds are sufficient for the intermediates to survive long enough to influence biological processes [212], and light-harvesting chlorophyll pigments can enable mammalian mitochondria to capture photonic energy and produce ATP [213]. It is interesting to note in this context that the ionizing radiation-induced bystander effect (RIBE) seems to be triggered by light emission during the return to the ground state of excited molecules, resulting in certain situations in a block of complex 1 in the mitochondrial electron transport chain (ETC) [214]. In the retina, rhodopsin captures light, and it is thought that the energy requirements for vision involve quantum tunneling in complex 1 of the mitochondrial ETC. In this context, the light harvesting is also suspected to underlie the so-called “magnetic compass” allowing bird and insect migrations to be so accurate.

- Cellular Respiration

Outside of the light-harvesting functions, respiration in mitochondria is thought to involve non-trivial quantum effects including entanglement, superposition, and tunneling [57]. Tunneling has recently been demonstrated to occur in complex 1, as noted above, but is likely to occur also in other complexes involved in electron transfer [162]. These processes lead to very efficient electron transfer, and it has been speculated that aging or certain degenerative diseases involving chronic fatigue and related loss of energy may involve defects at the quantum level [215]. Furthermore, the role of infrared (IR), at 700 nanometers to 1 mm, and its manipulation in influencing quantum behavior, especially within contexts such as the electron transport chain (ETC) [216] or its direct effects on water [217,218], emphasizes the significance of IR in modulating quantum processes within cellular environments. It is emitted by objects above absolute zero and is absorbed by water molecules, and it exerts a range of biological effects, including its influence on cellular respiration, gene expression, and cell signaling. Quantum effects may significantly mediate the biological impacts of IR (e.g., quantum entanglement can enable long-distance IR-mediated signaling, overcoming physical barriers between cells). Quantum tunneling also plays a role in the transfer of IR-mediated energy, allowing effects to propagate through barriers [219,220].

- Mutation/repair in DNA

One of the major areas of research in quantum biology concerns DNA replication, mutation, and repair. The key idea here is that, for replication to occur with minimal effort, the energy required to break a hydrogen bond between AT or CG base pairs can be reduced if the base pairs enter a superposition tautomeric state during which they tunnel [221,222]. However, when in the tautomeric state, they may be more prone to mutations. The Löwdin theory of mutation, published in 1963 and formalized in 1965, provides a convincing, if theoretical, discussion of how mismatch and missense mutations as well as deletions could be accounted for by this theory [223,224]. Quantum events (mainly entanglement) are also postulated to explain the ability of restriction endonucleases to coordinate breaks in the complementary strands of DNA to enable repair processes to occur.

- Cell signaling after energy capture/deposition

During the last thirty years or so, it has become increasingly apparent to radiobiologists that irradiated cells communicate with unirradiated cells and transmit a “memory” of the irradiation to their progeny [225]. While the occurrence of these events is not now disputed, no fully acceptable mechanism has emerged. This is particularly true of attempts to explain how non-clonal mutations emerge in the distant progeny of irradiated cells, or how cells or even organisms that are not targeted by radiation and show no ionizing radiation energy deposited in them can display all the signs of having been exposed, including chromosomal mutations, the upregulation of their repair capacity, mitochondrial changes, and cell transformation [225]. Theories involving quantum biology have not yet been advanced in this field, but many of the processes involved are thought to involve quantum behaviors. These include signaling, which involves ion gradients, and mitochondrial the ETC, the emission of UVA photons within which triggers release of exosomes that are captured by bystander cells and somehow lead to the ETC complex 1 block, as mentioned above [226,227]. The ETC collects electrons from NADH or FADH2 and transfers them through a series of electron carriers within multiprotein respiratory complexes (complex I to IV) to oxygen, generating an electrochemical gradient [228]. Their involvement extends to various diseases, including cancer, Alzheimer’s disease, and Parkinson’s disease [229,230]. As mentioned above, infrared radiation influences the electron transport chain (ETC) [231] in various ways (e.g., exciting electrons within the ETC complexes [232], leading to increased electron transport and ATP production, or enhancing the fluidity of the mitochondrial membrane, optimizing the efficiency of electron transport). IR radiation directly impacts water molecules within biological systems [233], leading to increased rotational and vibrational energy. IR can also break hydrogen bonds between water molecules, resulting in changes to water structure and dynamics.

Many of these processes may incorporate non-trivial quantum phenomena, including tunneling, superposition, and entanglement [32]. Notably, neurotransmitters and their inhibitors, such as serotonin, L-deprenyl, nicotine, ondansetron, or reserpine, possess the capacity to modulate or interfere with these forms of communication [109]. Tunneling mechanisms enable ions to traverse polarized cell membranes, offering a far more efficient means of generating bioelectric fields compared to classical diffusion [199,234]. Moreover, the concept that bioelectric fields could exist in a superposition of states is intriguing, as it could enhance the transmission of information in a more efficient manner than classical fields [235], which might play a crucial role in information processing within the brain, facilitating the brain’s ability to concurrently process data from various sources [236]. Additionally, the possibility that bioelectric fields might be entangled opens up the potential for long-distance communication, which could be instrumental in how neurons interact with one another and with other cells in the body [237,238]. Tunneling may also be integral in the generation of action potentials in neurons, which are rapid changes in the electrical potential facilitating neuron-to-neuron communication [234].

### 3.3. Anywhere Response Is Coordinated

The second major suite of biological actions that may involve quantum-level mechanisms is those where synchronization occurs at a level where classical mechanisms cannot explain the observations or where nonlinearity and thresholds predominate in the mechanism.

- Mitochondrial action

Mitochondria, like chloroplasts, are microbial-like organelles that many biologists believe were “captured” by primitive eukaryotic cells and harnessed to perform specific functions [239]. Respiration and photosynthesis are prime processes thought to involve quantum biology, as discussed above, but other functions of mitochondria may also involve quantum processes. For example, water interfaces in the mitochondrial membrane capture red–near-infrared (R-NIR) energy, and this quantum-like process is suggested to be responsible for the ability of low-level light therapy (LLLT) to energize people [240,241]. The synchronization of the biological functions of mitochondria may involve entanglement, explaining how the proton pump function in complex 1 can be associated with the reduction of quinone by NADH, even though several nanometers may separate them [241,242]. Mitochondria, the powerhouses of the cell, are central to cellular respiration and many other cellular functions, responsible for energy production, contain their own DNA, and are believed to have evolved from bacteria [243,244].

Quantum biology considerations extend to the role of cryptochromes within mitochondria, which may influence processes such as energy capture and electron transfer [245,246]. It is worth noting that cryptochromes are involved in the responses to blue and ultraviolet-A (UVA) light, playing a role in synchronizing biological functions. The generation and manipulation of triplet states in cryptochromes, initiated by photon absorption, are of particular interest in understanding the quantum mechanisms driving mitochondrial action [247]. Emerging evidence suggests that mitochondria communicate with each other through photons, thereby influencing various cellular processes [110]. This inter-mitochondrial photon communication contributes to the coordination of mitochondrial function, ultimately impacting overall cellular health. Chromophoric networks, comprised of molecules capable of absorbing and transferring light energy, play a pivotal role in these quantum processes. Notable examples include tryptophan and the aromatic networks found in biological systems [248,249]. One fascinating quantum effect within chromophoric networks is tryptophan in microtubules (e.g., super-radiance), where molecules collectively emit light, resulting in brighter emission compared to individual molecules [250]. This super-radiance phenomenon has the potential to enhance the efficiency of energy transfer processes in mitochondria [251,252]. How does super-radiance play a role in boosting the mitochondria’s energy capture and electron transfer processes? In addition to this, Herbert Fröhlich proposed that quantum effects influence biological systems through coherent vibrations, where all chromophores in a network vibrate in phase [252]. These coherent vibrations might extend to water molecules within mitochondria and could play a role in energy transfer and signal transduction [251], including plant leaves and nerve cells, which implies that these coherent vibrations may contribute to the energy transfer processes within mitochondria [253,254,255,256], further bridging the gap between established biology and the quantum world. The participation of cryptochromes, known for their sensitivity to blue and ultraviolet-A light, in processes related to energy capture and electron transfer within these organelles is important. Cryptochromes have the potential to synchronize and influence biological functions, which is crucial in understanding the quantum mechanisms that drive mitochondrial action [245,246].

- Tissue/organ/organism-level response

Related areas to cellular signaling discussed earlier include emergent responses at the level of the tissue and organ and tissue coordination via the nervous system. These can be reduced to functions of fundamental processes such as electromagnetic activity or electron transport involved in enzyme activity, DNA replication, ion-gated membrane channels, and, of course, energy production (photosynthesis and respiration), which have all been shown to involve quantum chemistry [257]. Interesting questions concern decision making at the tissue or organ level. For example, in irradiated cells, are there critical dose thresholds where apoptosis, which is a low-dose response to eliminate damaged cells, might be prevented so that organ function can be maintained even if compromised? Alternatively, are there damage-sensing mechanisms that inform cells on the best course of action to preserve tissue function? Are consciousness and psychopathological behavior emergent properties of the quantum brain? While such ideas are largely speculative, they are actively being discussed in quantum biology and quantum neurobiology [258,259]. The influence of coherent vibrations in water molecules, particularly influenced by Fröhlich’s ideas, could potentially play a role in various biological processes at this scale, which might include the coordination of tissues and organs through electromagnetic activity, electron transport involved in enzyme activity, DNA replication, and even consciousness. The quantum aspects of decision making within cells and the coordination of tissues become fascinating topics.

- Quorum sensing

Quorum sensing is a phenomenon first described in bacteria that refers to the ability to detect and respond to changes in population density by regulating gene expression [260]. Cryptochromes are sensitive to blue light and have the ability to generate and manipulate radical pairs upon photon absorption, potentially contributing to synchronizing the functions of social organisms, including both bacteria and animals [208,261]. Beyond their involvement in population-level communication, alongside chemical signaling, electrical signaling plays a vital role in the quorum sensing process [262,263]. In bacteria, a certain threshold number of individuals are required to trigger a gene expression change. It was thought that signals could only be chemical molecules, but, recently, evidence has emerged for electrical signaling [260]. This led Majumdar and Pal in 2017 to question whether quantum mechanics could explain the long-distance synchronization of functions in, for example, biofilms [264]. The function of potassium ion-gated channels, which are important in density-related signaling in a wide range of species, is also suggested to involve two entangled K ions. A further area where quorum sensing is suspected to involve quantum biology is in the aggregation of slime molds. These exist as single-celled amoeba-like organisms, but, when food is short, they respond to a variety of chemical or electrochemical signals and aggregate into a multicellular, differentiated form to produce fruiting bodies, the spores of which remain dormant until food is again plentiful.

- Population-level communication (bacteria/social animals)

Quorum sensing is a specific subtype of communication and signaling within and between cells and organisms. The field has been reviewed recently by Matarèse et al., 2020 [12,265], looking at chemical and physical signaling across the plant, microbial, and animal kingdoms and reviewing the evidence for acoustic signaling across the kingdoms of living organisms. Direct experimental evidence for quantum biological effects in social organisms as opposed to subcellular processes is scarce, but the whole organism-level communication mechanisms involving ion-gated channels and electrical or electrochemical gradients can be explained more easily by postulating the involvement of quantum processes [32]. One experiment that may involve quantum entanglement but could also be explained as a post-conditioning effect is described in Mothersill et al., 2018 [266]. The authors and others demonstrated that if irradiated fish swam with unirradiated fish, the unirradiated group upregulated a suite of proteins that were protective against radiation damage [267]. However, in this series of experiments, the fish met before one group was irradiated and did not meet again. Both groups of bystander fish (those meeting before and those meeting after their partners were irradiated) induced protective responses and bystander signaling.

### 3.4. When Quantum Behavior Goes Wrong

Stressors, ranging from chemicals to radioactive agents, impact living organisms [268,269,270]. This influence spans from the induction of cancer to the perturbation of hormonal systems and to the induction of hormesis and adaptive responses, reflecting the wide range of effects that stressors can impose [271,272,273]. At the heart of this intricate interplay lie quantum processes, particularly those of energy transfer and electron transport [32]. The effects of stressors on molecular processes have impacts on ecosystem health and its delicately balanced dynamics. These quantum phenomena are correlated to the core metabolic pathways that govern the behavior of countless organisms within ecosystems, which is critical in efficiently harnessing life-sustaining oxygen and vital nutrients across a wide range of ecological niches.

#### 3.4.1. Influences on Quantum Behavior: A Glimpse into Dynamic Responses

The complex relationship between quantum behavior and external influences in biological systems provides profound insights into how organisms dynamically adapt to their constantly changing environments.

- Temperature Variations

Temperature variation exerts a remarkable influence on quantum behavior and its impact on coherence originates from the kinetic energy and motion of particles. When temperatures deviate from the optimal range, increased thermal energy can disrupt the precise phase relationships required for coherence by causing particles to move randomly, which can disrupt the delicate balance of forces that hold the particles together in a coherent state [274,275,276]. If we increase the temperature of the electrons, they will start to move around randomly, which will disrupt the phase relationships between the electrons, and the beam will become incoherent. The same principle applies to other types of particles, such as photons and atoms. Consequently, energy transfer processes and cellular communication are affected, impacting the functionality of biological systems [277]. Moreover, temperature plays a significant role in quantum tunneling, since higher temperatures amplify the chances of particles overcoming energy barriers, thereby expediting reactions that rely on this quantum phenomenon. Conversely, lower temperatures hinder particle motion, potentially impeding effective energy barrier penetration [278,279,280]. This temperature-dependent quantum behavior carries tangible biological implications in which biological reactions dependent on quantum tunneling, such as crucial enzymatic processes for metabolism, can either accelerate or decelerate due to temperature variations. Organisms become more susceptible to stress during extreme temperature fluctuations, with cold or heat stress disrupting the delicate balance of biological reactions, with the consequence of metabolic pathways being impacted, jeopardizing the overall health of ecosystems [281].

- Electromagnetic Field Fluctuations

Electromagnetic fields, whether natural (e.g., lightning and solar flares) or human-made (power lines and electronic devices), introduce disturbances that interfere with quantum states and coherence, which disrupt the delicate phase relationships maintained by quantum entities, altering their coherent behavior and impacting energy transfer processes by causing the particles to absorb or emit photons or by causing the particles to scatter [282,283,284,285,286,287]. When a particle absorbs a photon, it jumps to a higher-energy state; when it emits a photon, it jumps to a lower-energy state; and when a particle scatters out of an electromagnetic field, it changes its direction of travel. All these transitions can also disrupt the phase relationships between the particles, leading to decoherence. Microwaves can disrupt the coherence of electrons in photosynthetic complexes, which can reduce the efficiency of photosynthesis and lead to cell damage; radio waves can disrupt the coherence of water molecules in cells, which can lead to a variety of health problems, including headaches, fatigue, and insomnia; while X-rays and other ionizing radiation can damage DNA by disrupting the coherence of electrons in DNA molecules, which can lead to cancer and other diseases [216,288]. Furthermore, electromagnetic fields impact quantum superposition in biological systems, influencing particle behavior and introducing uncertainties in the quantum states of biological molecules [57], which result in a disruption in coherence and energy transfer processes, leading to significant biological consequences, potentially causing inaccuracies in cellular communication and disrupting metabolic pathways [289]. Moreover, the perturbation of quantum superposition within biological molecules introduces uncertainties that might disrupt their intended functions [57,290,291]. When the quantum superposition of a biological molecule is perturbed, it can cause the molecule to collapse into one of its possible states, which can prevent the molecule from performing the functions that are associated with its other states. The perturbation of quantum superposition can also introduce errors into biological processes (e.g., if a protein is in a superposition of states that correspond to two different binding sites, then the perturbation of superposition could cause the protein to bind to the wrong site), which could lead to the malfunctioning of the protein and the biological process that it is involved in.

- Chemical Interactions

Chemical interactions, especially in environments containing stressors or reactive compounds, can introduce perturbations that specifically challenge the coherence of quantum systems, thereby impacting energy transfer processes and cellular functions. This occurs because chemical interactions have the potential to modify the energy landscapes that particles traverse during quantum processes, potentially altering the rates and outcomes of these reactions (e.g., the crucial regulatory mechanism governing various cellular processes, known as redox control, primarily operates through electron transfer—a quantum process—and can be significantly influenced by chemical interactions) [58,292]. Another illustrative example lies in the charge potential across cell membranes, which arises from the movement of ions across the membrane—a quantum process. The charge potential, commonly referred to as the membrane potential, is a result of ions migrating across cell membranes, and this phenomenon entails ions moving from regions of higher concentration to regions of lower concentration, engendering an electric current. However, this movement simultaneously generates a voltage across the membrane, counteracting the ions’ motion. When this voltage reaches an equilibrium value, the flow of ions ceases. Additionally, the cytoskeleton functions as a dynamic network of filaments, providing structural support while actively participating in various cellular processes, including the movement of ion channels and the response to alterations in the charge potential. These processes, like all physical phenomena, are fundamentally rooted in the principles of quantum mechanics. How is this a quantum process? At its core, all matter is governed by the laws of quantum mechanics, and this includes the behavior of ions traversing cell membranes. Quantum mechanics elucidates their conduct, encompassing energy levels and interactions (e.g., it elucidates how hydrogen ions (protons) are capable of passing through specific biological structures during processes like photosynthesis and cellular respiration). Nevertheless, although these processes fundamentally align with quantum mechanics, they are typically described and comprehended at the biological level through the lens of classical physics. The term “quantum processes” is generally reserved for situations explicitly involving or displaying observable quantum effects, which is not commonly the case for ion movement across cell membranes. To dive deeper into the quantum characteristics of these processes related to non-targeted effects during radiation, refer to our Proposed Model and discussion section.

- Radiation Exposure

Non-ionizing radiation, including visible light, ultraviolet light, and radio waves, lacks the energy to ionize atoms but still exerts biological effects. In contrast, ionizing radiation, such as cosmic rays and radioactive particles (e.g., X-rays, gamma rays, and alpha particles), profoundly challenges the coherence of biological systems. It disrupts the carefully maintained phase relationships of quantum particles, leading to alterations in their coherent behavior, with consequences extending beyond the quantum realm to impact cellular processes and overall organismal health [293,294]. Ionizing radiation can eject electrons from atoms and molecules, generating charged ions (via direct and secondary ionization). These ions disrupt the phase relationships between adjacent quantum particles and break nucleotide bonds in DNA, potentially causing mutations. The collision of charged ions with quantum particles disrupts their phases, leading to decoherence and scrambled phase information. Furthermore, ionizing radiation causes water molecules to undergo radiolysis, producing hydrogen and reactive hydroxyl radicals. These radicals damage DNA and other cellular components, altering the cell’s energy landscape and impacting quantum particle phase relationships. Additionally, hydrogen atoms from water radiolysis can diffuse through cell membranes, leading to the formation of new molecules with different chemical properties, further affecting the cell’s energy landscape and quantum particle phase relationships. Furthermore, ionizing radiation extends its impacts oxidative phosphorylation— a vital process that converts food into energy within mitochondria, which rely on quantum tunneling within enzyme-catalyzed electron transfer reactions. The deleterious effects of radiolysis-generated hydroxyl radicals inflict damage mitochondrial enzymes, interfering with quantum tunneling and oxidative phosphorylation, leading to a reducion in cellular energy production, with adverse consequences.

Finally, the Earth’s magnetosphere shields against cosmic radiation, but some particles can penetrate, especially during space travel, necessitating protective measures. The coherence disruption from radiation exposure affects energy transfer processes, cellular communication, and metabolic pathways, potentially leading to genetic abnormalities, cancer, impaired immune responses, and developmental anomalies, with far-reaching consequences for individual organisms and ecosystems [292,295].

#### 3.4.2. Low-Dose Mechanisms

The area of low-dose radiobiology is often described as a “wicked problem” in the sense that it involves complexity and uncertainty and thus no solution may be found until one emerges. Among the phenomena seen in the low-dose range (here defined as <0.5 Gy) are low-dose hypersensitivity (HRS), adaptive responses and hormesis resulting from direct energy deposition in cells or organisms, and NTE, including lethal mutations, genomic instability, and bystander effects, which result from signaling processes that activate changes in progeny or neighbors. These phenomena have been discussed in detail in the literature [296]. Here, we discuss whether these effects are related and suggest that quantum processes may underlie at least some of the steps in their manifestation. Of the first three processes, hormesis and adaptive responses are clearly related and an adaptive response is simply a form of hormesis that involves an inducible protective response due to low-dose exposure, which protects against further irradiation. At first glance, HRS might not appear to be related, but HRS manifests as a discontinuity in the low-dose part of the dose–response curve where the initial relationship changes abruptly, and the final slope of the curve is more resistant than the initial slope [297,298]. This more resistant part of the curve results from induced radioresistance (IRR) and involves the induction of repair systems when the dose exceeds a threshold in the region of 0.1 Gy. This phenomenon is probably also a form of hormesis but one that requires a threshold dose of radiation to be experienced in order to activate it. These direct effects of radiation have all been linked to increased ROS [22], probably resulting from the increased mitochondrial activity needed to generate energy for repair induction and execution. Thus, quantum biology is central to each process. However, what of the non-targeted effects, which are the subject of this paper? Genomic instability, one of the underlying “hallmarks of cancer” [299], is generally seen as an adverse effect leading to the unpredictable production of a variety of non-clonal lethal and non-lethal mutations [296]. However, it is very important to consider what an adverse effect is. This depends on the level at which “harm” or benefit is being assessed. Harm to an individual (cell or organism) is beneficial if it removes damage from the population. Harm in the form of high morbidity in a population may be beneficial if it drives selection towards a new, fitter phenotype. Lethal mutations are a sub-class of genomic instability that manifest as non-clonal lethal chromosomal damage, which, being lethal, could not have been induced by the initial ionizing radiation [296]. Genomic instability is now known to be driven by persistent high levels of oxidative stress [300], suggesting again that ROS underlies the generation of these non-clonal mutations. This leaves the bystander effect. Thought initially to be an adverse effect of low-dose exposure, radiation-induced bystander effects (RIBE) are now known to result in adaptive as well as deleterious effects. What outcome is documented again depends on the level of organization at which “harm” or “benefit” is being assessed. Recently, it has become clear that all the various effects demonstrated in vivo may reflect an ongoing inflammatory response to the initial radiation-induced injury that, in a genotype-dependent manner, has the potential to contribute to primary and/or ongoing damage displaced in time and/or space from the initial insult [301,302,303]. There is also direct evidence that non-steroidal anti-inflammatory drug treatment reduces such damage in vivo [304]. Wright’s group [305] took this further and demonstrated a clear difference in the inflammatory response to radiation depending on the genotype of the mice that were exposed. Bystander signaling was implicated in this mechanism. On the other hand, there is considerable evidence of anti-inflammatory processes being induced. This is well documented in the low-dose and hormesis context, as reviewed in the literature [8,306]. It does not appear to have been documented as a direct consequence of a non-irradiated organism receiving bystander signals. A review of the literature aimed at answering the question of whether low doses stimulate the immune response also produced many contradictory findings [307]. This again points to the complexity of low-dose and non-targeted responses, where many competing processes are in play. Whatever the result, there is no doubt that ROS and mitochondrial biochemistry are involved in the signaling mechanisms, meaning that quantum processes may underlie both low-dose responses and RIBE. However, there is an intriguing question—given that UVA biophotons can transmit the ionizing radiation-induced “information” through a plastic flask to unexposed cells in a different flask, and given that exosomes harvested from the cells exposed only to the UVA biophotons can induce RIBE in a further flask of unexposed cells, does this mean that UVA biophotons rather than ionizing radiation per se generate RIBE? This would have profound implications for our understanding of low-dose radiobiology and also for our efforts to understand or ameliorate the effects of low-dose exposures. UVA causes its cellular effects by generating ROS [308,309], so ROS-generated exosomes could be reprogramming cells to respond as a population to ionizing radiation.

#### 3.4.3. Quantum Effects in Radiation

The interaction of high-energy particles or photons, such as X-rays or gamma rays, with biological systems can indeed initiate a series of quantum events within irradiated cells. Two key quantum effects, tunneling and entanglement, are often highlighted in these discussions [57]. ROS are generated as byproducts of oxidative phosphorylation in the mitochondria or via cell signaling-induced NADPH oxidases in the cytosol. These ROS are fundamentally important as second messenger signaling molecules in cell biology and physiology as they play significant roles in cellular communication by transferring functional proteins, metabolites, and nucleic acids to recipient cells. The quantum behavior of ROS influences electron transfer and energy state transitions (e.g., the superoxide anion radical (O_2_^•−^) and hydrogen peroxide (H_2_O_2_), generated by the mitochondrial electron transport chain and by more than 40 enzymes, mainly NADPH oxidases, are regulated by growth factors and cytokines) [310,311,312]. Quantum mechanics offers a more comprehensive framework for understanding these interactions compared to classical physics. One notable example is the role of chiral molecules, distinguished by their unique geometric properties, in enhancing the efficiency of energy transfer processes through quantum tunneling. This inherent chirality is a critical feature of numerous biological molecules, such as nucleic acids and proteins, especially in the context of ionizing radiation [313,314]. The intringuing phenomenon of chiral-induced spin selectivity (CISS) effect, where the spin direction of electrons passing through chiral molecules influences the manifestation of ionizing radiation-induced quantum effects [315]. This interaction of chiral molecules with ionizing radiation is similar to other biological molecules. However, their unique geometric asymmetry contributes to specific interactions that further influence the quantum events that follow [316]. The CISS effect can facilitate long-range electron transfer in chiral molecules through mechanisms involving quantum coherence, tunneling, or entanglement [317]. Quantum processes, particularly those induced by radiation, can directly affect the bioelectric field (e.g., voltage-gated ion channels within irradiated cells) [318], which can lead to alterations in cellular membrane potential, creating a transformed electrochemical environment. The interactions between CISS and quantum tunneling in irradiated cells can significantly influence energy state transitions in chiral molecules [319,320] and this alteration of membrane potentials in non-targeted cells and the subsequent release of signaling molecules contained within exosomes is a complex process. Exosomes are known to play significant roles in cell-to-cell communication by conveying active biomolecules to target cells [321,322]. However, specific research linking this process directly to the alteration of membrane potentials in non-targeted cells is a new concept. ROS are key signaling agents generated by various cellular processes [311,323] and known to play significant roles in cellular communication by transferring functional proteins, metabolites, and nucleic acids to recipient cells. While ROS are known to influence signaling molecules [324], the quantum nature of the ROS influencing these signaling molecules is a concept that requires further exploration and validation in the scientific literature. The activation of various cellular responses in neighboring non-irradiated cells by the released signaling molecules is a well-documented phenomenon that includes the activation of cellular pathways, changes in gene expression, and modifications in cellular behavior [325], and these responses contribute to what is known as non-targeted effects in radiobiology. While these processes are individually recognized, the direct link between all these processes is not explicitly established in the literature.

## 4. Proposed Model and Discussion

**Figure 5:**
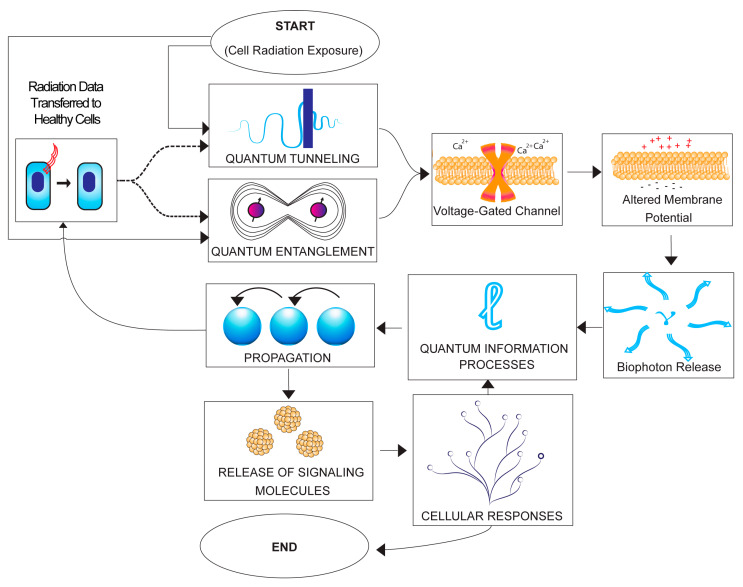
Quantum model for NTE induced by ionizing radiation. Hypothetical sequence: (1) ionizing radiation triggers quantum events involving tunneling and entanglement within irradiated cells; (2) quantum processes impact voltage-gated ion channels, altering cellular membrane potentials; (3) altered potentials lead to photon emissions, possibly carrying quantum-encoded information; (4) quantum information in emitted photons transfers to non-irradiated cells via non-targeted effects; (5) non-irradiated cells undergo quantum events, modulating ion channels and membrane potentials; (6) altered potentials prompt signaling molecule release from non-irradiated cells; and (7) released molecules induce cellular responses, including adaptive hormetic and adverse effects depending on context, contributing to observed NTE.

In this section, we provide a conceptual model, shown in Figure 5, that combines quantum biology concepts with the processes underlying radiation-induced non-targeted effects (NTE). We combine the fascinating features of quantum tunneling, entanglement, voltage-gated ion channels, photon emissions, and calcium fluxes in this model, which set the scenario for us to explore the complex mechanisms that eventually lead to the release of signaling molecules and the formation of NTE.

### 4.1. Hypothetical Sequence of Quantum Events in Radiobiology

- Ionizing Radiation Exposure: Ionizing radiation triggers quantum phenomena, including quantum tunneling and entanglement, within irradiated cells, which involve electron transfer and energy state transitions (e.g., reactive oxygen species (ROS); chirality and CISS effect; bioelectric field alteration).
- Cellular Impact: Quantum processes influenced by radiation-induced quantum phenomena affect the activation of voltage-gated ion channels within irradiated cells, which leads to changes in cellular membrane potentials, creating an altered electrochemical environment.
- Photon Emission: Altered membrane potentials in irradiated cells can result in the emission of photons, potentially carrying encoded quantum information that propagates through the cellular microenvironment.
- Quantum Information Transmission: The quantum information encoded in emitted photons is transmitted to neighboring non-irradiated cells through non-targeted effects, facilitated by signaling molecules or physical interactions between cells.
- Quantum Events in Non-Targeted Cells: Non-targeted cells that receive quantum-encoded information may undergo quantum events similar to those in irradiated cells. These events can influence the opening and closing of voltage-gated ion channels, further impacting cellular membrane potentials [318,326]. The precision and specificity of the quantum-encoded information within emitted photons, particularly those involved in CISS, can enhance these events [327].
- Signaling Molecule Release: The alteration of membrane potentials in non-targeted cells leads to the release of signaling molecules contained within exosomes. These signaling molecules, enriched by the quantum properties of ROS and the quantum-encoded information, activate various cellular responses in neighboring non-irradiated cells. These responses encompass the activation of cellular pathways, changes in gene expression, and modifications in cellular behavior, contributing to the observed non-targeted effects (NTE) in radiobiology.
- Cellular Responses: Released signaling molecules induce cellular responses in neighboring non-irradiated cells, activating cellular pathways, altering gene expression, or modifying cellular behavior, which contribute to the observed non-targeted effects (NTE) in radiobiology.

### 4.2. Quantum Tunneling in the Activation of Voltage-Gated Ion Channels

We propose that the phenomenon of quantum tunneling, where particles surpass classical energy constraints and overcome potential barriers, plays a role in ionizing radiation-triggered quantum tunneling within irradiated cells, possibly involving electron transfer and energy state transitions. This connection between quantum tunneling and ion channels, which regulate ion flow across cell membranes, suggests that tunneling may influence the activation of voltage-gated ion channels, facilitating cellular communication and signaling pathways critical in non-targeted effects (NTE). Ionizing radiation can generate free radicals, damage DNA, cause protein denaturation, and disrupt cell membranes, creating potential barriers that ions can tunnel through, which may play a role in the biological effects of ionizing radiation. In addition to these examples, it is also possible that ionizing radiation can induce tunneling in other ways, such as by interacting with the electrons in cells. However, more research is needed to understand the specific mechanisms by which ionizing radiation induces quantum tunneling in cells.

Ion channels, integral membrane proteins that allow ions to flow across cell membranes, play a pivotal role in cell signaling, muscle contraction, and nerve conduction by enabling the flow of ions into and out of cells, which subsequently triggers various signaling cascades, including those responsible for muscle contraction, nerve conduction, and hormone release. In muscle cells, ion channels facilitate the entry of calcium ions, leading to muscle fiber contraction, while, in nerve cells, these channels regulate the flow of sodium and potassium ions, generating electrical signals that propagate along nerves [328,329,330,331]. It has been proposed that quantum tunneling might influence certain ion channel functions (e.g., may contribute to the rapid movement of ions through ion channels) [326,332,333]. Specifically, voltage-gated ion channels, which serve as crucial regulators in cellular communication, open and close in response to shifts in the cell membrane’s electrical potential. The subtle energy barriers encountered by ions during channel opening are navigated through the tunneling process. This process, in turn, alter cell electrical activity, influencing the release of signaling molecules. One way that quantum tunneling could affect the activation of voltage-gated ion channels is by allowing ions to pass through the closed channel. This would be possible if the channel has a narrow region that acts as a potential barrier. The ions could tunnel through this barrier, even if they do not have enough energy to overcome it classically. Another way is by affecting the conformational changes that occur when the channel opens and closes. These conformational changes involve the movement of different parts of the channel protein. Quantum tunneling could allow these movements to occur more easily, which could lead to faster and more efficient channel activation. Additionally, the concept of quantum tunneling introduces an intriguing possibility in which signaling molecules might traverse cell membranes even without precise alignment (e.g., cells must communicate across distances without physical contact, such as the immune system’s capacity to detect pathogens). Moreover, the concept of encoding quantum information through particle movement (e.g., electrons, protons, and photons) introduces a novel dimension to cellular communication, promising unexplored pathways for information transfer that could be tested experimentally by measuring the opening probability of voltage-gated ion channels in irradiated cells. If quantum tunneling plays a role in NTE, we would expect to see an increased opening probability of voltage-gated ion channels in irradiated cells. Investigating the role of quantum tunneling in the activation of voltage-gated ion channels is a logical extension of the quantum biology model, and experimental evidence supporting this prediction could highlight a quantum mechanism at play in cellular responses to radiation. However, it may be challenging to disentangle quantum tunneling effects from other factors influencing ion channel behavior.

### 4.3. Quantum Entanglement in the Release of Signaling Molecules

We propose that exosomes, DAMPs, and cytokines could be responsible for transmitting quantum-encoded information between cells, potentially influencing the bystander effect. Key players in this process, exosomes travel through the bloodstream and lymphatic system, impacting cell behavior and cargo delivery [321,322]. Damage-associated molecular patterns (DAMPs) and cytokines, crucial in cellular communication during environmental stressors like radiation exposure, may also involve quantum components in their signaling processes [334,335]. Exosomes can significantly influence recipient cells by transferring their cargo, potentially including microtubules that support quantum entanglement. Additionally, DAMPs have the ability to initiate and perpetuate an immune response, potentially generating ROS. Growing evidence suggests that both reactive oxygen species (ROS) and exosomes may be associated with quantum processes. ROS, chemically reactive oxygen-containing molecules, are natural byproducts of oxygen metabolism, playing important roles in cell signaling and homeostasis. However, during environmental stress, ROS levels can dramatically increase, potentially leading to cell damage and oxidative stress. Notably, changes in membrane potential in irradiated cells can lead to the emission of photons encoded with quantum information [336] and both, exosomes and DAMPs have been found to contain biophotons, considered to play a role in quantum communication with quantum entanglement involvement. Furthermore, cytokines have exhibited interactions with electromagnetic fields, hinting at possible quantum properties. If exosomes, DAMPs, and cytokines indeed play a role in transmitting quantum-encoded information between cells, strategies could be developed to target these molecules and block the bystander effect. One method to explore the impact of exosomes, DAMPs, and cytokines on biophoton emission from irradiated cells is to conduct experiments involving irradiating a cell culture, collecting exosomes, or assessing the DAMP and cytokine levels in the culture medium. Subsequently, the culture conditions can be manipulated by introducing exosomes, a DAMP-blocking substance, or a cytokine-inhibiting agent, depending on the specific experiment. Finally, the emitted biophoton levels from the culture can be measured. This approach allows for the examination of these cellular components’ roles in biophoton emission, which may vary with different radiation sources and cell types, and their potential impact on the bystander effect. Developing strategies to target exosomes, DAMPs, and cytokines to mitigate the bystander effect holds promising implications in terms of advancing cancer treatment, managing inflammation in chronic diseases, enhancing immune function, reducing radiation exposure risks, and deepening our understanding of quantum communication in cellular processes. Ultimately, this research may lead to the development of innovative diagnostic and therapeutic tools in the field of biology and medicine.

Quantum entanglement enables particles to communicate across extended distances, revolutionizing intercellular communication. Exosomes, vital mediators in cellular communication, could leverage quantum entanglement to facilitate long-distance signaling between cells. This advancement allows communication to occur over significantly greater distances than previously considered possible. Quantum entanglement enhances the precision of exosome targeting by utilizing inherent correlations and non-local properties, delivering signaling molecules such as DAMPs, cytokines, and ROS with remarkable precision directly to specific target cells, thereby improving drug delivery accuracy. Furthermore, quantum entanglement introduces the possibility of signaling molecules in exosomes penetrating cell membranes more efficiently than classical fluctuations. Quantum tunneling, a quantum effect defying classical energy constraints, enables entangled molecules to traverse cell membranes with ease, enhancing the efficacy of cargo delivery within cells. The entanglement between signaling molecules within exosomes fosters coordinated signaling between cells, enhancing their overall response to environmental changes like radiation exposure. In contrast to classical fluctuations, which offer only local and random correlations, quantum entanglement has the potential to provide a higher level of organization and synchronization in cellular responses.

### 4.4. Quantum Information Transmission and Cellular Impact

There is further evidence that suggests that biophotons emitted by cells may encode quantum information, as indicated by their chiral polarization [337,338,339], fluctuations [340], and ability to act as signaling agents [104,341]. Here, we propose that the quantum information encoded in emitted photons is a mode of communication between irradiated and non-irradiated cells. This transmission is facilitated by signaling molecules or physical interactions between cells. When designing experiments to test the role of biophotons in non-target and bystander effects, it is important to control for factors that could affect biophoton collection (e.g., disrupt their quantum properties), gene expression (e.g., temperature and light exposure), and other cellular processes. To test the hypothesis that entangled biophotons convey information about radiation damage to other cells, a cell culture may be exposed to radiation, measure biophoton emission from irradiated and non-irradiated cells (photomultiplier tubes (PMT) or avalanche photodiodes (APD)), compare characteristics (such as their intensity, wavelength, or polarization), and repeat with different cells and radiation types. To test the hypothesis that quantum fluctuations in biophotons influence cellular responses to radiation damage, a cell culture should be exposed to radiation and one could measure the gene expression (such as quantitative real-time PCR (qPCR) or RNA sequencing), treat it with a substance that disrupts quantum fluctuations (such as strong magnetic fields or lasers), measure the gene expression again, and compare it to untreated cells, repeating with different cell types and radiation types. To test the hypothesis that biophotons emitted by irradiated cells act as signaling agents that affect gene expression and other cellular processes, a cell culture should be exposed to radiation, and one should collect the emitted biophotons, treat another cell culture with the collected biophotons, measure the gene expression, and compare it to untreated cells, repeating with different cell types and radiation types.

### 4.5. Quantum Coherence in Cellular Signaling

We propose that quantum coherence could enhance the efficiency and flexibility of voltage-gated ion channels in cellular communication, allowing them to navigate multiple pathways simultaneously. This hypothesis is grounded in the concept of superposition, a fundamental aspect of quantum coherence, as described in previous sections. It suggests that ion channels, by existing in multiple states simultaneously, could explore different signaling routes concurrently, resulting in faster and more efficient cellular communication. This novel hypothesis challenges our understanding of cellular signaling and is supported by experimental evidence, including the observation of quantum coherence in biophotons emitted by living cells. If validated, this concept could revolutionize our approach to diseases caused by disruptions in cellular signaling and potentially lead to the development of innovative therapies. The intriguing possibility of superposition-driven ion channel conduct holds the potential to improve cellular communication efficiency and interaction flexibility. It could expedite cellular responses to environmental changes, such as metabolic adjustments in response to temperature fluctuations, by allowing voltage-gated ion channels to traverse various routes simultaneously. Experimental investigations could aim to detect quantum coherence in signaling pathways. The detection and estimation of quantum coherence have become feasible through the development of various methods, including the construction of coherence witnesses for finite-dimensional states [342] or the extraction of coherence witnesses directly from experimental data obtained through two-pulse pump–probe spectroscopy [343]. Additionally, the concept of the incoherent witnessing of quantum coherence has emerged as a proof-of-principle protocol that utilizes entangled probes to detect the presence of quantum coherence [344]. Several platforms have been explored to conduct experiments, such as the use of linear optics, nuclear magnetic resonance, and superconducting systems [343,345]. Quantum coherence plays a vital role in advancing various quantum detection and control techniques (e.g., in the realm of coherent anti-Stokes Raman scattering (CARS) spectroscopy, quantum coherence is a fundamental element that has led to significant progress in multiple pathway quantum beat spectroscopy [346]).

### 4.6. Supporting Evidence

While there is limited experimental evidence correlating quantum processes and non-thermal effects (NTE), several studies have shown promising results in this regard. Mathematical models and in vitro experiments have demonstrated that quantum processes can indeed influence the behavior of voltage-gated ion channels and the release of signaling molecules [347]. For example, one study indicated that quantum tunneling can increase the opening probability of voltage-gated ion channels, thereby altering the electrical activity of cells [326]. There is another study that presented some evidence of quantum coherence affecting the selectivity and transport of ion channels [332]. Moving beyond theoretical models and in vitro findings, animal studies have provided additional support for this concept, with one noteworthy study demonstrating that quantum coherence could offer cellular protection against radiation-induced damage, suggesting that quantum coherence potentially plays a role in repairing damaged cells [348]. Additionally, an intriguing in vivo experiment involving fish provided evidence of an entanglement-like effect in the communication between irradiated and unirradiated fish, manifesting as correlated changes in the quantum states of particles within and between cells [266]. Quantum phenomena may have important effects on cellular processes (e.g., quantum tunneling may make cells more sensitive to radiation, potentially raising the risk of cancer, or quantum entanglement may improve the coordination of cellular responses to different types of stress by enhancing the secretion of signaling molecules through exosomes).

### 4.7. Expanding the Model to Explore Factors Shaping Non-Thermal Effects of Radiation

Diverse forms of radiation, varying in energy levels and interaction mechanisms with biological entities, significantly influence NTE. For example, the distinction between X-rays and gamma rays introduces subtleties in NTE manifestation [61,349]. X-rays, with higher energy, can potentially induce cancer-causing DNA damage. In contrast, gamma rays, interacting primarily with electrons, tend to cause non-DNA damage, such as oxidative stress and inflammation, leading to distinct aspects of NTE [350,351,352,353]. The radiation dosage emerges as a crucial determinant of NTE severity, in which higher doses increase the likelihood of NTE, while even low doses can induce NTE, especially in susceptible individuals. The radiation dosage also dictates the nature of NTE, with high doses resulting in acute NTE like radiation sickness and lower doses associated with chronic NTE such as cancer development [354]. Considering the impact of the radiation type on the quantum phenomena within cells involves exploring how different radiation types affect quantum tunneling and the entanglement of particles such as electrons; additionally, an individual’s characteristics, including genetic variations, age, sex, and overall health, significantly influence NTE susceptibility (e.g., children, due to ongoing cellular development, are more vulnerable to radiation-induced NTE). Similarly, individuals with underlying health conditions exhibit heightened susceptibility [61,354,355,356,357]. The adaptation of the quantum biology model could include incorporating radiation-specific quantum tunneling and entanglement events. Tailoring cellular responses to quantum processes based on individual characteristics offers a more comprehensive framework that could be experimentally tested by exposing various cell types to different radiation types and doses, measuring the resulting NTE. Differences in NTE patterns among cell groups based on the radiation type, dose, and individual characteristics would validate this prediction, which could serve as a starting point for future research in the field. Expanding the model to encompass a holistic framework, considering the intricate interplay between quantum processes and biological systems, could capture the complexity of quantum biology and its implications for NTE. 

In recent years, there has been growing interest in the potential role of quantum effects in communication and homeostasis. Communication and homeostasis are fundamental mechanisms that maintain life, enabling cells to coordinate their activities and respond to environmental changes, while homeostasis maintains the delicate balance of internal conditions necessary for survival. Quantum phenomena like entanglement, photons, and coherence could play significant roles in improving the efficiency, robustness, and long-range communication necessary to maintain homeostasis [358]. What is the role of quantum biology in communication and homeostasis, specifically in the brain? How can microtubules and Posner molecules mediate quantum entanglement and coherence? How can these phenomena be used for intercellular signaling and the emergence of consciousness in the context of non-targeted radiation effects? Microtubules, hollow rod-shaped structures distributed throughout cells, including the brain, participate in a variety of cellular functions, like cell division, intracellular transport, and signaling [359,360], and might facilitate quantum entanglement within different brain regions due to their properties conducive to entanglement, such as their long, slender structures and ability to conduct electricity, enabling long-range coherence [361,362,363]. These microtubules may form a quantum network supporting non-local communication and potentially influencing consciousness emergence. Photons, carriers of electromagnetic radiation, can serve as efficient messengers between cells. In the brain, photons could enable inter neuronal signaling across synapses. Furthermore, field-based consciousness theory posits that consciousness is a universal field present throughout the universe, which suggests that consciousness is not limited to the brain but rather is a ubiquitous phenomenon [364]. Microtubules, found throughout the body, might be integral to field-based consciousness by enabling long-distance communication within a quantum network. This, in turn, raises the possibility of consciousness extending beyond the confines of the brain. Posner molecules, responsive to electromagnetic radiation, exist in living cells, including the brain, and could be involved in non-targeted radiation effects, where non-ionizing radiation can disrupt their function and cause DNA damage [259,360,365].

### 4.8. Quantum Concepts in Radiobiology and Quantum Computing

Quantum information holds the potential to revolutionize our understanding of biological processes, particularly in the context of the non-targeted effects (NTE) of ionizing radiation. Here, we explores the encoding and transmission of quantum information within cellular systems and its implications for NTE and discusses the practical applications of quantum concepts in the field of radiobiology. Quantum information, carried by photons or molecular vibrations, could traverse cellular microenvironments, transcending traditional biochemical pathways. It has the potential to impact intercellular communication, shedding light on non-locality in NTE patterns. In the realm of quantum computing, qubits are foundational, allowing superposition and entanglement. In quantum biology, the introduction of “Qdits”, multi-level computational units (see Figure 3), could revolutionize biological data representation and processing. Qdits may elucidate the mechanisms underlying the NTE of radiation exposure, with the potential to target specific DNA damage pathways involved in NTEs. Qdits could enhance bioimaging techniques, capturing dynamic changes in biological molecules and cells in response to radiation exposure, potentially enabling the more precise imaging of molecular and cellular changes during NTE and aiding in the identification of new biomarkers for NTE. This could have significant implications in cancer diagnosis and therapy, improving the accuracy in identifying at-risk individuals and monitoring treatment responses. In terms of practical applications, researchers have used the twin-field quantum key distribution (QKD) protocol to enable secure quantum communication over a record 605 km of fiber. For the first time, this study expanded the range of fiber-based quantum communications beyond 600 km. The recently created twin-field QKD protocol could bypass the distance constraint, but it will require new methods to be used with fiber lengths greater than 500 km [366,367,368]. Coherence is one of the key factors that allows quantum communication to work in optical fibers. Quantum communication relies on the ability to transmit and manipulate quantum states of light, which are very delicate and can be easily disrupted. Fibers provide a relatively stable environment for the transmission of quantum light, but even small disturbances can cause the qubits to lose their coherence. Another important factor is that the fibers must be very transparent to the wavelengths of light that are used for quantum communication. This is because quantum light is typically very weak, and even small losses can make it difficult to detect the signal at the other end of the fiber. Applying these concepts to NTE, exosomal signaling molecules and emitted biological photons could both convey quantum-encoded information, showing that quantum processes could alter intercellular reactions in unexpected ways. While water is a transparent liquid that can transmit light, it absorbs light at certain wavelengths, making it unsuitable for long-distance quantum communication. Similarly, blood, which contains light-absorbing red blood cells, is not a suitable medium for quantum communication. In contrast, neurons, nerve cells that transmit signals throughout the body, contain microtubules, hollow tubes capable of transmitting light and supporting quantum entanglement, making them a potential candidate for in-body quantum communication. Quantum communication within the body could occur through the transmission of entangled photons between neurons. Entangled photons are quantumly linked, so that measuring the state of one photon instantly reveals the state of the other photon, even if they are physically separated. This could allow neurons to communicate with each other in a way that is not possible with classical communication. Nonetheless, numerous challenges must be addressed before quantum communication can be experimentally realized within the body or harnessed for its enhancements. One challenge is that microtubules are very small and delicate, so it would be difficult to manipulate them in a way that is necessary for quantum communication. Another challenge is that the body’s immune system would likely attack any foreign objects that were implanted in the body, such as quantum devices.

### 4.9. Future Research and Experimental Directions

These fields are in their early phases and will require more study to properly comprehend the function of quantum processes in NTE. Improving the model’s credibility and advancing experimental methods (e.g., high-resolution imaging and quantum state analysis) might give more precise data; conducting control tests under different conditions would validate the repeatability of the reported entanglement-like phenomena. Furthermore, establishing the presence and understanding the underlying mechanisms of entanglement within this specific biological system are essential by focusing experiments on characterizing the correlations in quantum states within and across different cells (e.g., molecules, cellular structures, or signaling pathways contributing to entanglement-like states). Moreover, the manipulation and monitoring of these factors through experimental designs could reveal insights into the processes behind entanglement and its impact on cellular communication. Correlations between particles originating from nuclear reactions are necessary to investigate the existence and mechanisms of entanglement within NTE, and momenta, spins, and angular distributions should be meticulously examined to identify strong correlations that challenge classical explanations and hold the key to unravelling the nature of entanglement, along with its profound influence on the quantum states of particles in the NTE process. The ramifications of quantum processes for cellular communication are vast, and quantum communication holds the potential to revolutionize the speed and efficiency of cell-to-cell interactions, even spanning considerable distances, which could reshape disease treatment strategies, enabling targeted drug delivery and the precise targeting of cancer cells. Quantum biology is still in its infancy but its potential to improve cellular communication is certainly strong.

## 5. Conclusions

We have presented a conceptual model and discussion that combine the fascinating characteristics of quantum effects, which reveal fundamental connections that may provide answers to long-standing biological puzzles. Indeed, we intended to uncover the basic principles driving cellular communication with quantum physics at the convergence with biology, with the examination of quantum phenomena including coherence, entanglement, and tunneling in non-targeted effects in radiation biology. We now face a turning point that represents a significant advancement in radiation research as we start to understand these quantum events paving the way to enhanced radiation therapy, improved radiation safety protocols, and improved environmental well-being and sustainability.

## Data Availability

All the relevant data and information for the review are present within the review itself.
